# Optimal selection for an air suspension system on buses through a unique high level parameter in genetic algorithms

**DOI:** 10.1016/j.heliyon.2022.e09059

**Published:** 2022-03-04

**Authors:** Truong Manh Hung

**Affiliations:** Department of Automotive Mechanical Engineering, Faculty of Mechanical Engineering, University of Transport and Communications, Hanoi, 100000, Viet Nam

**Keywords:** Vehicle dynamics, Air suspension, Air spring, Genetic algorithm, Ride comfort, Road holding

## Abstract

Air suspension systems are being widely used on ground vehicles in general and buses in particular. The element that makes the superiority of this system is air spring. This paper introduces the application of genetic algorithms method to optimize the parameters of the air spring element. A full model of a bus using air suspension system with air spring element, which is modeled based on the Gensys model. From the real experiments, the bounds of seven optimized parameters of the air spring are determined. In which, by using a single *α* value, it is possible to determine the optimal parameters according to each desired criteria between the road safety and the ride comfort. Optimal results are verified in the time domain simulations with random road profile according to the ISO standard 8608. The results show that by using the optimal parameters of the air spring when *α* = 0.5, the ride comfort is improved about 15% while the road safety is still guaranteed.

## 1 Introduction

### 1.1 Background

In recent years, automobile suspension systems with pneumatic spring (air suspension system) have become increasingly popular in passenger cars, buses, high-comfort vehicles, trucks and heavy vehicles. Due to the superiority of the ride comfort and the road holding, the current air suspension system has gradually replaced the conventional suspension system using metal spring such as leaf springs or torsion bars, etc [1]. The use of air suspension system for buses is done mostly for new buses manufactured from 2015 to the present. Since the air spring element of this system is able to change the stiffness due to the compressive properties of air in the air spring element, the active or semi-active control methods can be applied on this system to be suitable for different motion modes [2, 3].

The decisive factor to the superior properties of the air suspension system is the air spring element. Therefore, previous studies focused on manufacturing research and testing to determine the appropriate air springs for each type of vehicles. The air spring is located between the sprung mass and unsprung mass, each of which is linked to the auxiliary air tank and the high-pressure air flow feed [4]. Initial studies on air suspension systems only dealt with the air movement between the air springs and the auxiliary air tank. However, this approach does not produce outstanding efficiency for vehicles with large variable loads. Therefore, the solution of using a levelling valve was given, so far it has been widely applied on trucks and buses. Depending on the load and movement conditions, the levelling valve will change the internal pressure of the air spring [5]. The research to control and optimize the parameters of the suspension system in general and the air suspension system in particular is the goal to ensure the relationship between ride comfort and safety of movement of the vehicles. These are also topics of interest to many researchers [6, 7, 8, 9].

### 1.2 Related works

Typical models of the air spring element in the suspension system can be listed as follows: Vampire, Nishimura, Gensys, Simpac [10, 11]. In these models, the Gensys model is determined to have high accuracy with the actual air spring forms [12, 13]. Comparative studies between theoretical and experimental models have been conducted when fully connecting the air spring and the auxiliary air tank [14, 15]. In addition, in the studies with the aim of evaluating the force of the air suspension system, the authors also used a simple model [16, 17]. To evaluate the effectiveness of the air suspension system in improving the ride comfort and the road safety of buses, previous studies used ¼ model [18], ½ model [19] and full model [5]. There are a few studies using the full model with 7 degrees of freedom (DOF) but not including the driver and passenger models and studies focusing on measuring the signals on the experimental buses [5]. This raises a problem of vehicle dynamics as to whether it is possible to evaluate or optimize the parameters of the bus and the air suspension system when considering a full model of buses. Therefore, it is necessary to optimize the parameters of the air suspension system which is limited to air spring elements with the full model.

The genetic algorithm (GA) is a search method based on Charles Darwin's theory of natural evolution. This algorithm reflects the process of natural selection in which the healthiest individuals are selected to reproduce in order to produce the offspring of the next generations [20]. The application of GA method to find the optimal parameters related to vehicle dynamics has been carried out by many studies [21, 22]. For example, optimizing parameters of semi-active suspension control system [23], active anti-roll bar system [24], etc. The authors Nguyen et al [25] have applied this method in the study of the air suspension system applied on a semi-trailer vehicle when considering the road destruction criteria dynamic load coefficient (DLC). As for the air suspension system with air spring element installed on the bus, its characteristic is nonlinear with high complexity. Besides, when the bus model is fully considered, the determination of the objective functions to perform the reasonable optimization problem is also the basis for considering applying the genetic algorithm method in this study.

### 1.3 Paper contribution

In the author's previous research, the experimental studies were set up to determine the parameters of the air spring [26], the very valuable contributions of this manuscript are listed as follows:

- The multi-objective optimization problem is built with two main criteria: ride comfort and road holding. These are the two core criteria used in the study of vibration as well as in determining the suspension system on road vehicles.
- The most prominent element that makes up the superior characteristics of the air suspension system is the air spring element. This study optimizes 7 main parameters of air spring element by using genetic algorithm method through a unique high level parameter *α.* This study is one of the first to follow this potential approach.
- Experimental study determines the bound values of 7 parameters and applies the optimization problem to get the Pareto curve with the change of ride comfort and road holding. Research results when the speed of the bus is 80 km/h with the road profile in random form class C according to ISO 8608, have shown that by using the proposed method in genetic algorithm, it is possible to optimize the air suspension system on buses. This will open up potential applications in the design, manufacture and control of air suspension systems.

The structure of the paper is built as follows: Section 2 presents multi-objective optimization problem, Section 3 establishes a full vertical model of a bus using the air suspension system and the anti-roll bar system, Section 4 introduces the construction of the optimal problem in selecting the optimal parameters of the air spring by genetic algorithm, Section 5 introduces the simulation results and evaluation with the road profile in random form in the time domain. Conclusions and directions for further research are given in Section 6.

## 2 Multi-criterion optimization

GA has been and is being widely applied in the fields of science and technology. Its famous applications are finding optimal solutions to multi-objective optimization problems and are shown in Eq. (1). In which, *F(x)* is the objective vector, *x* the decision vector, *C* the set of the searching space *x*.

(1) $$\min_{x\in C}\;F\left(x\right)=\left[\begin{array}{l} f_{1}\left(x\right)\\ f_{2}\left(x\right)\\ \;\vdots \\ f_{n_{obj}}\left(x\right) \end{array}\right],\;n_{obj}..2,$$

In fact, all objective functions *f*_*1*_*, f*_*2*_ *... f*_*nobj*_ can be minimized by the existence of an ideal solution *x∗* being not highly feasible.

In order to solve the problem (1), there are a lots of formulations, for example: goal programming methods, weighted global criterion method, min-max method [27]. The weighted sum method is one of the most well-known and easy to understand solutions that convert a multi-objective problem into a single-objective. This study uses a specific case of the weighted sum method, in which the convex combination of the targets is returned for the multi-objective function vectors as:

(2) $$\min\;J=\sum_{i=1}^{n_{obj}}\alpha_{i}f_{i}\left(x\right),\;s.t\;x\in C,\;\sum_{i=1}^{n_{obj}}\alpha_{i}=1$$

**Figure 1:**
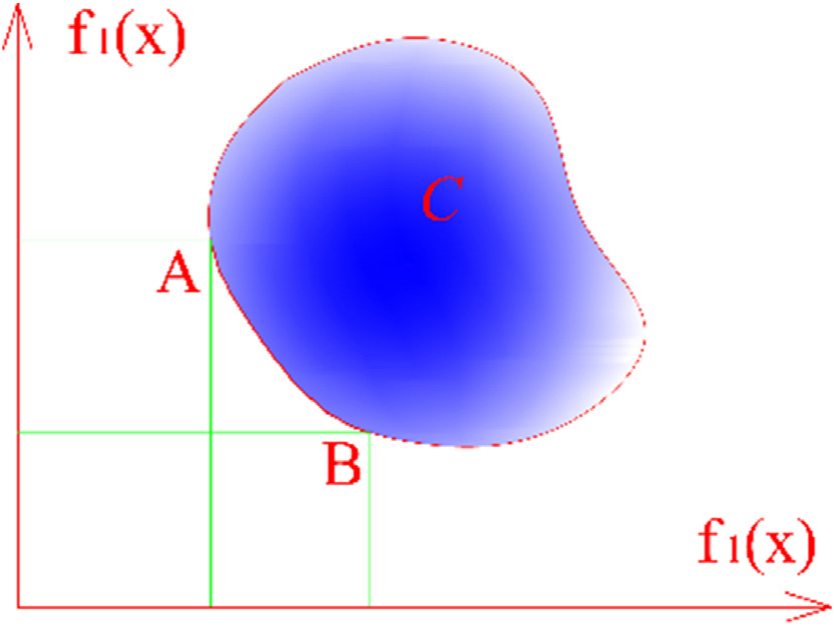
Pareto curve's description with two conflicting objective functions.

The gradient of function *J* is represented by the vector $\alpha =\left(\alpha_{1},\alpha_{2},...,\alpha_{n_{obj}}\right)$. Due to the use of various sets of *α*, several points in the Pareto can be created. The shape of the Pareto curve shows the nature of the trade-off between the conflicting objective functions. Figure 1 indicates an example of a Pareto curve, where all the points between A($f_{2}\left(\hat{x}\right),\;f_{1}\left(\hat{x}\right)$) and B($f_{2}\left(\tilde{x}\right),\;f_{1}\left(\tilde{x}\right)$) define the Pareto curve. Therefore, these points are called Pareto curve's non-inferior points [24].

## 3 Vehicle modelling

**Figure 2:**
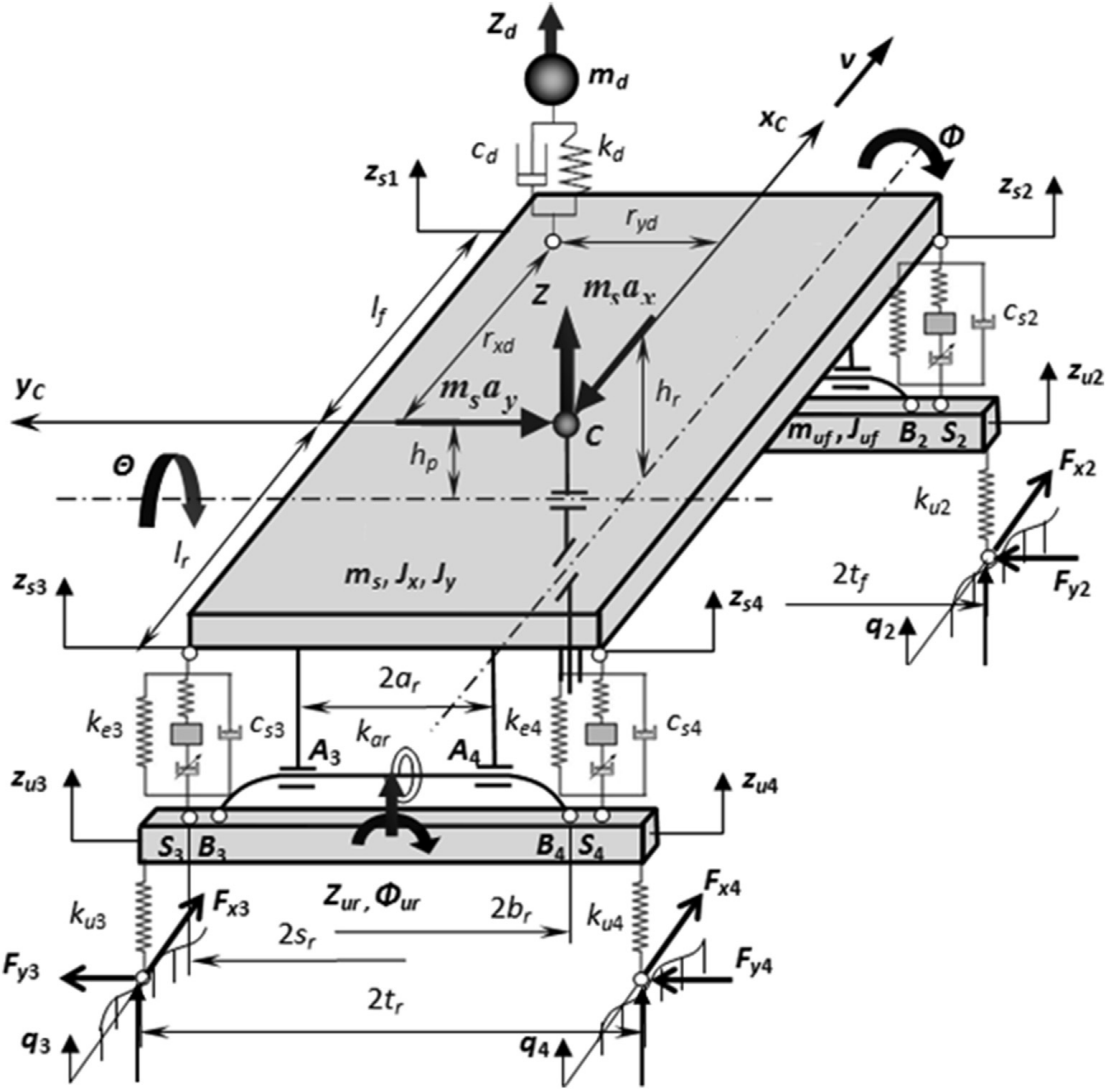
A bus model using air suspension and passive anti-roll bar systems.

In this section, the author introduces a bus model of 15 tons using the air suspension system on both axles as shown in Figure 2. This model has 8 DOF including 1 DOF for the driver model, 3 DOF for the vehicle body (sprung mass) and 4 DOF for the unsprung mass at the two axles. The state vector ***z*** includes the displacement of the driver *Z*_*d*_; vertical displacement, roll and pitch angles of the sprung mass *Z*, *Ф*, *Θ*; vertical displacements, roll angles of the unsprung mass *Z*_*uf*_, *Z*_*ur*_, *Ф*_*uf*_, *Ф*_*ur*_, which is characterized for the 8 DOF of the bus model. The dynamic equation of the bus model is determined in Eq. (3). The symbols and parameters of the bus model are shown in Table 1 [26].

(3) $$\left\{ \begin{array}{l} \text{ ​ ​}m_{uf}{\ddot{Z}}_{uf}\;=-\sum_{i=1}^{2}\left(F_{ui}-F_{si}\right),\\ \text{ ​}m_{ur}{\ddot{Z}}_{ur}\;=-\sum_{i=3}^{4}\left(F_{ui}-F_{si}\right),\\ \text{ ​}J_{uf}{\ddot{\Phi }}_{uf}\;=-\sum_{i=1}^{2}F_{ui}r_{uyi}-M_{af}+\sum_{i=1}^{2}F_{si}r_{syi},\\ \text{ ​}J_{ur}{\ddot{\Phi }}_{ur}\;=-\sum_{i=3}^{4}F_{ui}r_{uyi}-M_{ar}+\sum_{i=3}^{4}F_{si}r_{syi},\\ m_{d}{\ddot{Z}}_{d}\;=-F_{d},\\ \text{ ​}m_{s}\ddot{Z}\;=F_{d}-\sum_{i=1}^{4}F_{si},\\ \text{ ​}\left(J_{x}+m_{s}h_{r}^{2}\right)\ddot{\Phi }=F_{d}{r_{y}}_{d}+m_{s}a_{y}h_{r}+M_{af}+M_{ar}-\sum_{i=1}^{4}F_{si}r_{syi},\\ \text{ ​}\left(J_{y}+m_{s}h_{p}^{2}\right)\ddot{\Theta }=-F_{d}r_{xd}-m_{s}a_{x}h_{p}+\sum_{i=1}^{4}F_{si}r_{sxi}. \end{array}\right.\;$$

**Table 1:** Symbols and parameters of bus and air spring models.

Symbols	Description	Value	Unit
*h*_*r*_*/h*_*p*_	Height of CG of sprung mass from roll/pitch axis	0.7/0.5	m
*m*_*d*_	Driver weight	75	kg
*d*_*d*_	Damping coefficient of seat	1000	Ns/m
*k*_*d*_	Stiffness coefficient of seat	52538	N/m
*m*_*s*_	Sprung mass	13425	kg
*m*_*uf*_*/m*_*ur*_	Unsprung mass at the front/rear axle	663/1156	kg
*L*	Wheelbase	6.16	m
*t*_*f*_*/t*_*r*_	Distance of two wheels at the front/rear axle	2.05/1.86	m
*l*_*f*_*/l*_*r*_	Length of the front/rear axle from the CG	3.73/2.43	m
*y*_*d*_*/x*_*d*_	Length of the seat from pitch axis/CG	0.7/3.70	m
*I*_*x*_*/I*_*y*_	Roll/Pitch moment of inertia of sprung mass	18000/71692	kgm^2^
*J*_*uf*_*/J*_*ur*_	Roll moment of inertia of unsprung mass at front/rear axle	696.6/999.86	kgm^2^
*d*_*sf*_*/d*_*sr*_	Damping coefficient of front/rear suspension	11733/32804	Ns/m
*k*_*sf*_*/k*_*sr*_	Stiffness coefficient of front/rear suspension	303844/397007.7	N/m
*k*_*uf*_*/k*_*ur*_	Stiffness coefficient of front/rear tyre	793211/1586422	N/m
*s*_*f*_*/s*_*r*_	Length of front/rear suspension from unsprung mass CG	1.72/1.72	m
*k*_*af*_*/k*_*ar*_	Torsional stiffnesses of the anti-roll bar at front/rear axle	25000/35000	Nm/rad
*r*_*o*_	Density of compressed air	1.293	kg/m^3^
*n*	Polytropic index	1.3	
*p*_*a*_	Atmospheric pressure	100000	Pa
*A*_*e*_	Effective area		m^2^
*l*_*s*_	Air pipe length		m
*d*_*s*_	Diameter of air pipe		m
*A*_*s*_	Cross sectional area of air pipe		m^2^
*V*_*bo*_	Initial volume of air spring		m^3^
*V*_*ro*_	Initial volume of auxiliary air tank		m^3^
*β*	Exponent parameter		
*C*_*z1*_	Typical stiffness coefficient for air spring		N/m
*C*_*z2*_	Typical stiffness coefficient for auxiliary air tank		N/m
*b*_*z*_	Viscous damping		Ns/m

In Eq. (3), *F*_*si*_ are the forces of the air spring elements and the dampers and *M*_*ai*_ are the torques generated by the passive anti-roll bar systems at the two axles. These two elements (air spring and passive anti-roll bar) are defined as follows:

### 3.1 Air spring modelling

**Figure 3:**
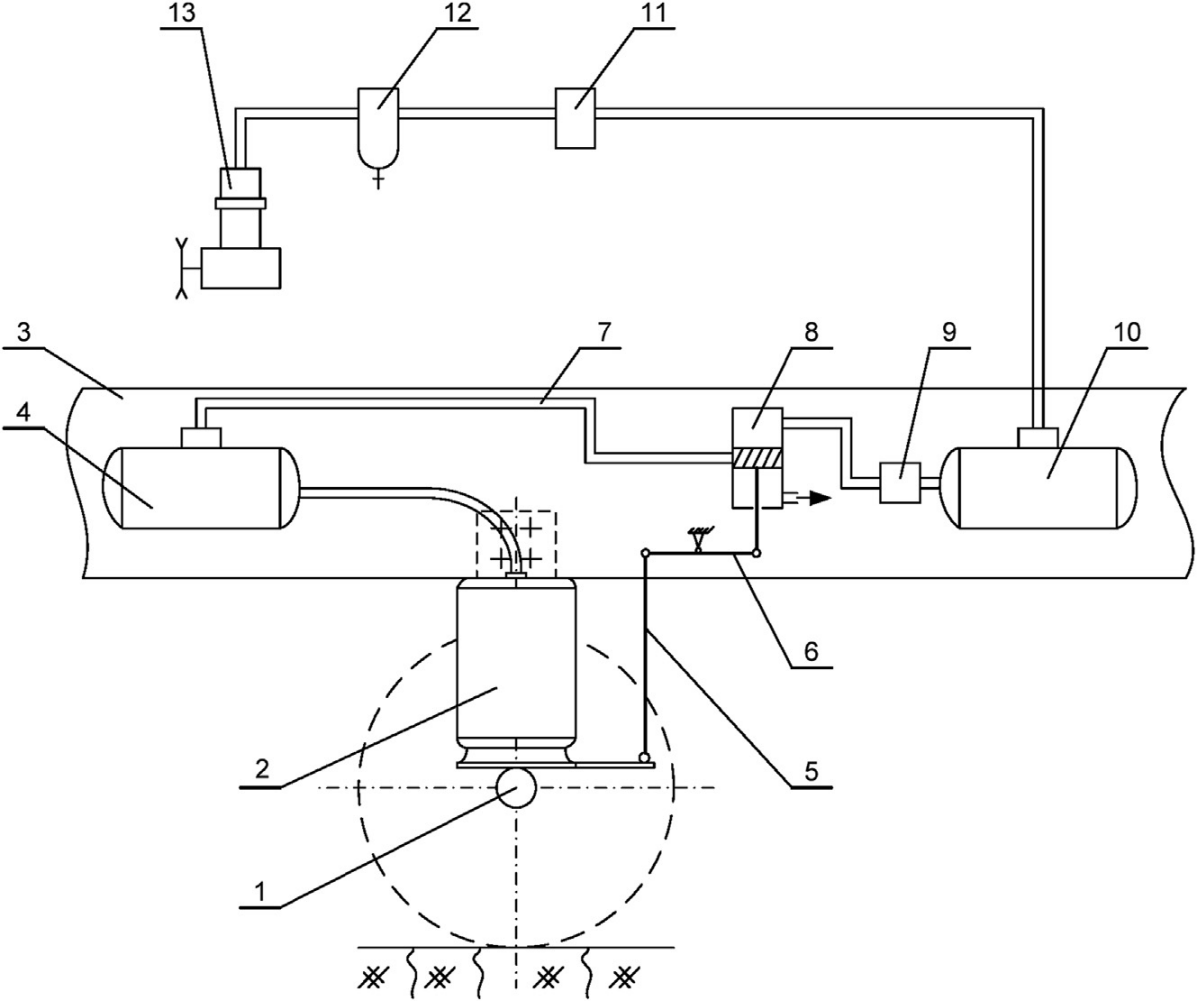
Air spring element layout.

The air spring element of the air suspension system on a bus is diagrammed as shown in Figure 3. In this diagram, the axle is denoted as 1, 2 is the air spring element, 3 is the chassis, 4 is the auxiliary air tank, 5 is the pull bar, 6 is the transom, 7 is the surge pipe, 8 is the levelling valve, 9–11 are the air filter tanks, 10 is the air tank, 12 is the oil and water drainer, 13 is the compressor. The role of the compressor 13 is to provide the high pressure to the air spring element. However, depending on the condition of the load and the quality of the road surface, the levelling valve 8 will change the pressure inside the air spring element. This is also a particularly important element of air suspension system compared to leaf spring suspension system, because by this way the stiffness of the spring element can be changed.

**Figure 4:**
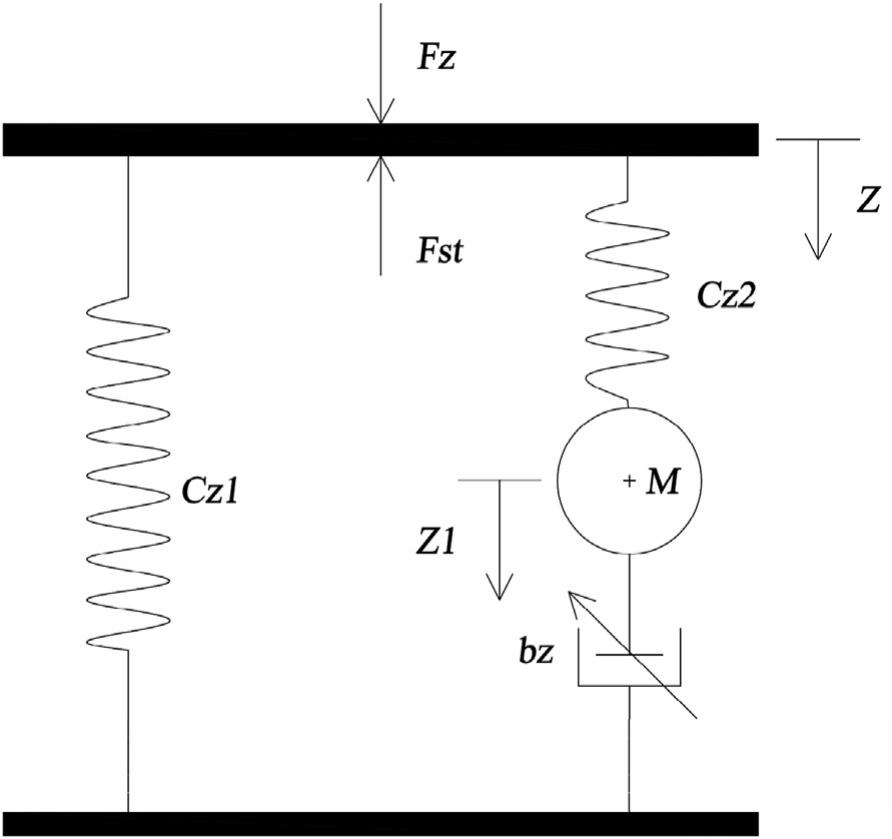
Gensys model for an air spring element [12].

**Figure 5:**
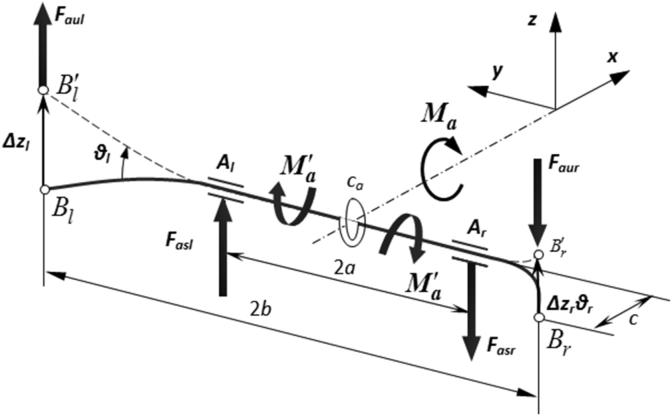
Diagram of the passive anti-roll bar system on bus model.

**Figure 6:**
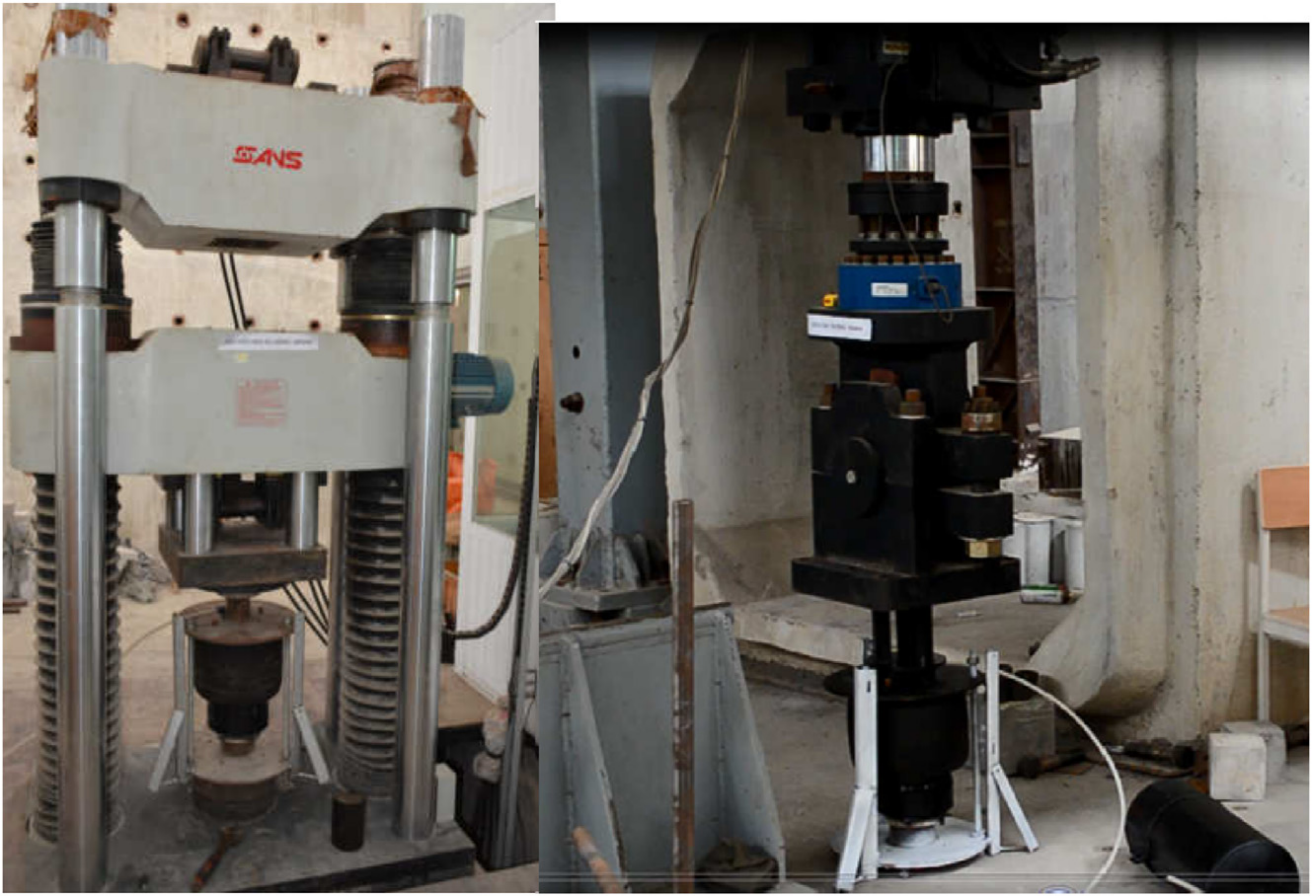
Experimental determination of air spring parameters' bounds.

In order to study the air suspension system, a number of studies have introduced different models of the air spring element such as Simpac, Vampire, Nishimura, Gensys, etc [10, 11]. However, many researchers have been interested in the Gensys model. Here, the Gensys model is used to build the fully-integrated bus model as shown in Figure 4 [12, 14, 26]. The dynamic equation of the air spring element is determined in Eq. (4) [12, 26]. The symbols of the air spring model are summarized in Table 1. Experimental equipment for air spring element and anti-roll bar structure diagram is shown in Figures 5 and 6.

(4) $$\left\{ \begin{array}{l} M{\ddot{z}}_{1}=C_{z2}\left(z-z_{1}\right)-b_{z}{\left|{\dot{z}}_{1}\right|}^{\beta }sign\left({\dot{z}}_{1}\right)\\ F_{z}=\left(p_{0}-p_{a}\right)A_{e}+C_{z1}z+C_{z2}\left(z-z_{1}\right) \end{array}\right.$$

In the previous study [26], the author presented an experimental study to determine the relative basic parameters of the individual air spring element. However, the problem is that when the bus is in motion, the criteria of the road safety and the ride comfort needs to be balanced, so the optimal selection of the parameters of the air spring element plays an important role. In the structure of the air spring element, the main parameters that need to be optimized including: Effective area *A*_*e*_, Air pipe length *l*_*s*_, Diameter of air pipe *d*_*s*_, Initial volume of air spring *V*_*bo*_, Initial volume of auxiliary air tank *V*_*ro*_, Exponent parameter *β* and Initial pressure inside the air spring *p*_*0*_.

### 3.2 Passive anti-roll bar modelling

The anti-roll torque generated by the anti-roll bars on the front axle *M*_*af*_ and the rear axle *M*_*ar*_ can be determined as follows: when the relative vertical displacement is different between the wheel and the vehicle body on the right and left at the same axle $\left(\Delta z_{r}\neq \Delta z_{l}\right)$, it will appear a ratio corresponding to the relative torsion angle of the anti-roll bar $\left(\theta_{l}-\theta_{r}\right)$. On the anti-roll bar, with the angular stiffness *k*_*a*_ [Nm/rad], there is a torque $M{´}_{a}\left[Nm\right]$ that resists the relative displacement between the wheels and it is determined as Eq. (5).

(5) $$M{´}_{a}=k_{a}\left(\theta_{l}-\theta_{r}\right)\approx k_{a}\frac{\Delta z_{l}-\Delta z_{r}}{c}=k_{a}\frac{\left(z_{sAl}-z_{uBl}\right)-\left(z_{sAr}-z_{uBr}\right)}{c}=F_{au}c$$

This torque will create reaction forces *F*_*au*_ [N] acting on the axle and *F*_*as*_ [N] acting on the vehicle body at the positions associated with the anti-roll bar:

(6) $$\begin{array}{l} F_{aul}=-F_{aur}=\frac{M{´}_{a}}{c}=\frac{k_{a}}{c^{2}}\left(\Delta z_{l}-\Delta z_{r}\right)=k_{au}\left[\left(z_{sAl}-z_{uBl}\right)-\left(z_{sAr}-z_{uBr}\right)\right]=\frac{k_{a}}{c^{2}}\left(\Phi -\Phi_{u}\right)b,\\ F_{asl}=-F_{asr}=F_{aul}\frac{b}{a}=k_{a}\frac{b}{c^{2}a}\left(\Delta z_{l}-\Delta z_{r}\right)=k_{as}\left[\left(z_{sAl}-z_{uBl}\right)-\left(z_{aAr}-z_{uBr}\right)\right]=k_{a}\frac{b}{c^{2}a}\left(\Phi -\Phi_{u}\right)a \end{array}$$

The stabilizing torque produced by the anti-roll bar is determined as follows:

(7) $$M_{a}=2F_{asl}a=2F_{aul}\frac{b}{a}a=2F_{aul}b=2k_{a}\frac{b}{c^{2}}\left[\left(z_{sAl}-z_{uBl}\right)-\left(z_{sAr}-z_{uBr}\right)\right]=2k_{a}{\left(\frac{b}{c}\right)}^{2}\left(\Phi -\Phi_{u}\right)$$

Concatenating equations from 3 to 7, the dynamical equations of the fully-integrated bus model using the air suspension system are summarized as follows:

(8) $$\left\{ \begin{array}{l} M\;\ddot{z}=K_{w}z_{1}+Q\;u+C\;\dot{z}+K\;z\\ M_{w}{\ddot{z}}_{1}=C_{z2}G_{sw}z-C_{z2}z_{1}-C_{\beta }{\left|{\dot{z}}_{1}-G_{uw}\dot{z}\right|}^{\beta }sign\left({\dot{z}}_{1}-G_{uw\left(4x8\right)}\dot{z}\right) \end{array}\right.$$

where the matrices *M, M*_*w*_*, C, C*_*β*_, *K, K*_*w*_*, C*_*z2*_*, G*_*uw*_*, G*_*sw*_, *Q* are presented in [26].

$z={\left[Z_{d},Z,\Phi ,\Theta ,Z_{uf},Z_{ur},\Phi_{uf},\Phi_{ur}\right]}^{\text{T}}$ is the state vector;

$z_{1}={\left[{z_{1}}_{1},{z_{1}}_{2},{z_{1}}_{3},{z_{1}}_{4}\right]}^{\text{T}}$ is the vector for the air flow volume;

$u={\left[q_{s}^{T},q_{u}^{T}\right]}^{T}={\left[0,\left(m_{s}a_{y}h_{r}\right),\left(-m_{s}a_{x}h_{p}\right),q_{1},q_{2},q_{3},q_{4}\right]}^{\text{T}}$ is the external disturbances.

## 4 Optimal parameter selection for an air spring by genetic algorithms

### 4.1 Optimization objectives

When a bus moves on the road, the two most concerned criteria that are the road safety and the ride comfort. However, these two criteria often contradict each other, that is, when the road safety is improved, the ride comfort tends to decrease and vice versa. Therefore, the author builds a multi-objective optimization problem based on these two criteria. The ride comfort criteria is characterized by the root mean square (RMS) value of the driver's acceleration as shown in Eq. (9). Meanwhile the road holding criteria is characterized by the root mean square value of the interaction force between the wheels and the road in the vertical direction in Eq. (10).

(9) $$RMS\left({\ddot{Z}}_{d}\right)=\sqrt{\frac{1}{T}\int_{0}^{T}{\ddot{Z}}_{d}^{2}\left(t\right)dt}$$

(10) $$RM\text{S}\left(F_{zd}\right)=\frac{1}{4}\left(\sum_{i=1}^{4}\sqrt{\frac{1}{T}\int_{0}^{T}F_{zdi}^{2}dt}\right)$$

Therefore, the objective function is selected as follows:

(11) $$f=\alpha f_{s\text{af}ety}+\left(1-\alpha \right)f_{\text{comf}ort}$$

where *α* is high level parameter only, represents the gradient of function *f* as Eq. (2). $f_{c\text{omf}ort}=RMS\left({\ddot{Z}}_{d}\right)$ and $f_{s\text{af}ety}=RM\text{S}\left(F_{zd}\right)\zeta$ are performance indices corresponding to the ride comfort and the road safety of the bus. Here the author needs to emphasize that the coefficient $\zeta =e^{-3}$ is used with the goal of reducing the difference in the magnitude of the two criteria between acceleration and force.

By setting the objective function as shown in Eq. (11), when the value of *α* moves 0, the optimal goal will be to find the parameters of the air spring to improve the ride comfort. Meanwhile, when the value of *α* moves 1, the optimal goal is to improve the road safety. It should be noted that the values of *α* is positive in the range from 0 to 1.

### 4.2 Multi-criterion optimization problem formulation

The basic characteristics of the air spring element can include: $C_{z1}=\frac{p_{0}nA_{e}^{2}}{V_{b0}+V_{r0}}$ is typical stiffness coefficient, $M=m_{k}{\left(\frac{A_{e}}{A_{s}}\frac{V_{r0}}{V_{b0}+V_{r0}}\right)}^{2}$ is typical mass of the circulating air flow, $b_{z}=\frac{1}{2}A_{s}r_{0}k_{T}{\left(\frac{A_{e}}{A_{s}}\frac{V_{r0}}{V_{b0}+V_{r0}}\right)}^{\beta +1}$ is viscous damping. Meanwhile, for the auxiliary air tank, it's typical stiffness coefficient $C_{z2}=\frac{p_{0}nA_{e}^{2}}{V_{b0}+V_{r0}}\frac{V_{r0}}{V_{b0}}=C_{z1}\frac{V_{r0}}{V_{b0}}$. The above characteristics are derived from the basic parameters of the air spring element, including: *l*_*s*_, *d*_*s*_, *β*, *A*_*e*_, *V*_*bo*_, *V*_*ro*_, *p*_*0*_. In order to perform the optimization problem, the author sets the variables for these parameters in Eq. (12). The selection of all these parameters for the optimization problem, allows the synchronous determination of parameters from measurable values such as length and diameter to relative values such as exponent parameter and effective area. This will ensure that the parameters of the most optimal air spring element are given in a perfect relationship with the bus model.

(12) *l*_*s*_*= p*_*1*_*; d*_*s*_*= p*_*2*_*; β = p*_*3*_*; A*_*e*_*= p*_*4*_*; V*_*b0*_*= p*_*5*_*; V*_*r0*_*= p*_*6*_*; p*_*0*_*= p*_*7*_

The Multi Criterion Optimization (MCO) problem for the optimal selection parameters of the air spring element can be determined as follows [24]:

(13) $$\begin{array}{l} \min_{p\in P}\;f\left(p\right),\;f\left(p\right):={\left[f_{safety}\;f_{\text{comf}ort}\right]}^{T}\\ P:=\left\{p={\left[p_{1},\;p_{2},\;p_{3},\;p_{4},\;p_{5},\;p_{6},\;p_{7}\right]}^{T}\in R|p^{l}\leq p\leq p^{u}\right\} \end{array}$$

where *f(p)* is the vector of objectives, *p* denotes the vector of optimal parameters, and *p*^*l*^*, p*^*u*^ represent the lower and upper bounds of the parameters' range.

The lower and upper bounds of the parameters’ range are given in Table 2. These limits have been measured and compared from a sample of an air spring during the experiment as shown in Figure 2.

**Table 2:** Lower and upper bounds of the air spring element.

	l_s_	d_s_	β	A_e_	V_b0_	V_r0_	p_0_
	p_1_	p_2_	p_3_	p_4_	p_5_	p_6_	p_7_
Lower bound	0.5	0.001	1	0.05	0.01	0.01	3000000
Upper bound	5.0	0.03	2	0.3	0.6	0.5	7000000

### 4.3 Genetic algorithm method for optimal parameter selection

GA is more and more applied since the first study in [28], and after that in the books [29, 30]. The influence of the algorithms has been proven in optimization in a variety of real-world applications, and they are improved upon by natural selection. They are the same as global optimization techniques that use probabilistic search, multiple points, random matching (crossover, mutation) and information of previous iterations to estimate and grow the population. Compared with other optimization methods, a significant advantage of GAs is that they search regardless of the nature of the objective functions and constraints.

GA initializes with a random set. Through genetic function: selection, crossover and mutation, a new population is formed. The healthiest individuals built from their physical values are selected using a process of selection, crossover, and mutation that is applied to create new populations. When the optimal criterion is satisfied or a certain number of generations are obtained, the genetic activity on the individuals of the population is maintained. The four basic concepts of the mechanism of action of the GA method are defined as follows: 1) Mutation; 2) Crossover; 3) Selection; 4) Fitness function [24, 28, 31]. In general, optimization solving algorithms converge to a local minimum. In Genetic Algorithm method, mutation is used to get out of this local minimum. Mutations are applied to some individuals of a generation. Usually, mutations will be bad and make the result worse and they will not be selected for the next generation, but sometimes, a mutation causes an individual to get close to a different (and sometimes better) local minimum. The higher the mutation rate is, the more 'space' will be searched and the higher the chance that the global minimum is found.

In this study, the optimal selection of the parameters of the air spring element is followed the process developed by Holland [28]. For this technique, the determination capacity for every individual is established in proportion to its respective fitness, through a roulette wheel. The mutation occurs with a small probability of 0.095, meanwhile the crossover occurs with a high probability of 0.9. Therefore, the proposed parameter optimization procedure for the air spring element is as follows:

Step 1 Start with the selected parameters as *p = p*_*0*_. This *p*_*0*_ value is also selected through the experimental results: l_s_ = 3.0; d_s_ = 0.0034; *β* = 1.8; A_e_ = 0.0875; V_b0_ = 0.028; V_r0_ = 0.0589; p_0_ = 400000 [26, 32].

Step 2 Choose upper, lower bounds; offset; scale factor; starting point shown in Table 2.

Step 3 Set up the optimal algorithm form, choose the target vector with the transformation value of α in the range [0, 1], then the minimization problem is solved.

Step 4 Screening individuals, using crossover and mutation methods to create the next generations: *p = p*_*new*_.

Step 5 Conduct a new generation assessment. If the specified criteria do not match, the problem moves to step 3 with *p = p*_*new*_; otherwise, the problem stops and saves the optimal individual: *p*_*opt*_ *= p*_*new*_.

By following the above five process steps, the optimal results will be obtained for each value of *α* according to the correlation between the two criteria of the road safety and the ride comfort.

## 5 Simulation results and analysis

### 5.1 Optimal parameters of air spring

**Figure 7:**
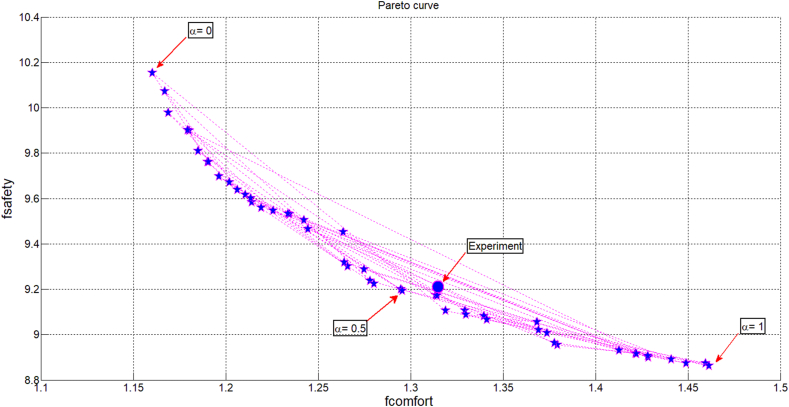
Pareto curve with some points of optimization results.

Performing simulation on Matlab/Simulink software with continuous execution for random generations, the Pareto curve shows the relationship between the two objective functions (Eqs. (9), (10), and (11)) obtained as shown in Figure 7. This result clearly shows that when the values of the parameters to be optimized run automatically on the whole bounds, the obtained optimal points have clearly formed the Pareto curve. When the *α* value moves zero the goal of improving the ride comfort is satisfied, and when the *α* value moves 1 the goal is to improve the road safety. Thus, the optimization problem has met the stated goal, through adjusting the unique *α* value, the objectives are rotated reasonably.

Corresponding to the value of *α*, the optimal set of parameters of the air spring element is determined. Normally, to create a rationality between the road safety and the ride comfort, the air spring element is designed and selected so that these two criteria are relatively harmonious, ie *α* value is from 0.3 to 0.7. In this study, the author is interested in an *α* value of 0.5. The optimal parameters of the air spring element when the value *α* is 0.5 is determined as follows: l_s_ = 2.9289 m; d_s_ = 0.0040 m; *β* = 1.5464; A_e_ = 0.1820 m^2^; V_b0_ = 0.3350 m^3^; V_r0_ = 0.3286 m^3^; p_0_ = 4.1652e+05 Pa.

### 5.2 Evaluation of optimal results with a random road profile

In this section, the parameter optimization results of the air spring element are evaluated in the time domain when the bus uses three types of suspension systems: leaf spring suspension (dash-dotted line), air suspension system with experimental parameters (dashed line), air suspension system with the optimal parameters of the air spring determined by the genetic algorithm method (solid line). The speed of the bus is considered at 80 km/h. Here the road profile is chosen according to ISO standard 8608 type C [26, 32, 33, 34]. In this scenario, the road profile signal fed to the right wheels is 1 s slower than the left wheels, to create lateral oscillations for the bus.

**Figure 8:**
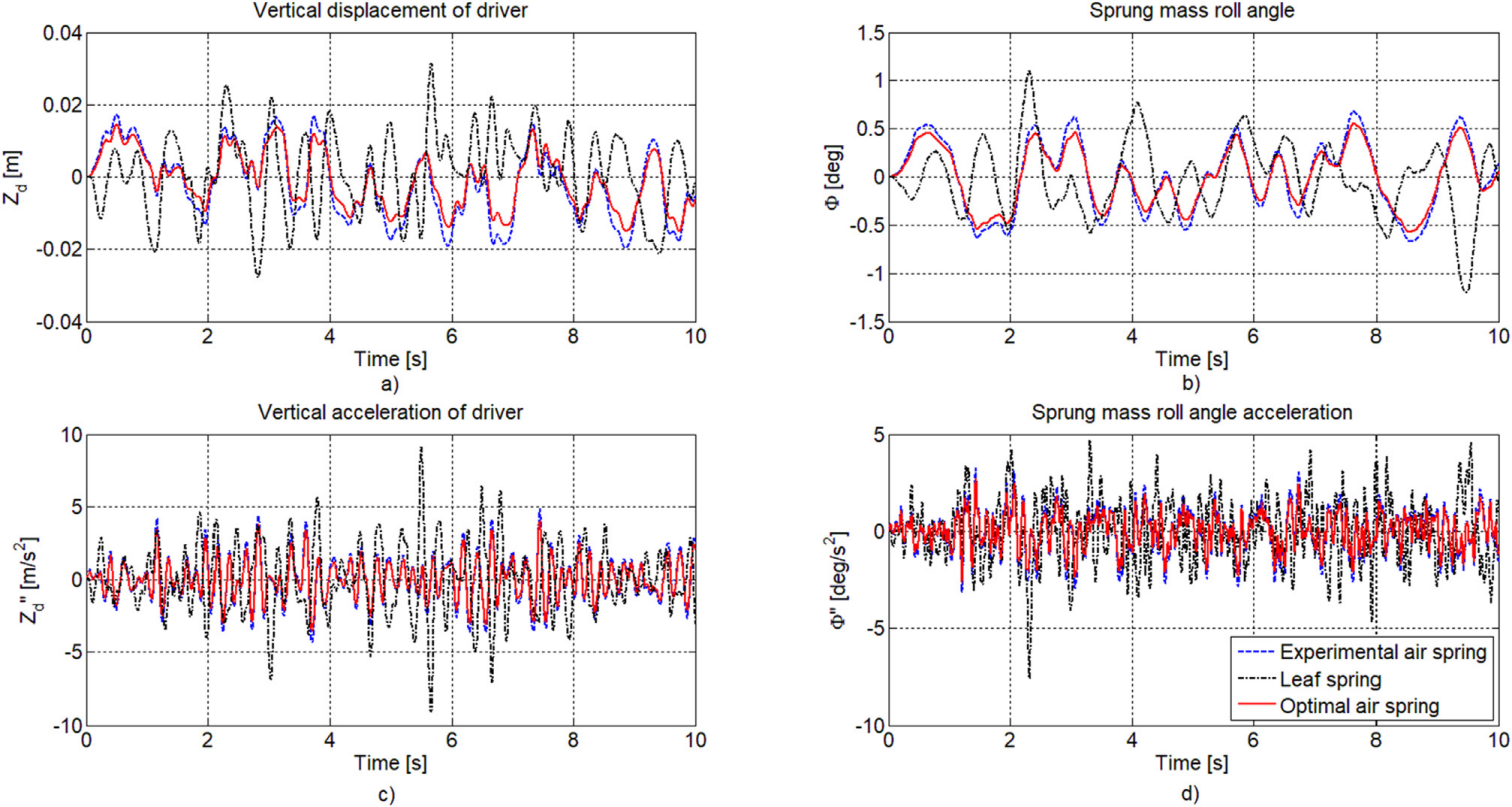
Time response of a) Vertical displacement of driver, b) Sprung mass roll angle, c) Vertical acceleration of driver, d) Sprung mass roll angle acceleration.

Figure 8 depicts the time response of the variables related to the ride comfort. It can be seen that the amplitude of the driver's acceleration in the case of using an air suspension system with optimal air spring element has been reduced by about 10%–15% compared to bus using an air suspension system with air spring element determined from experiment. Similar results are obtained for the vehicle's body displacement, roll angle and its acceleration. Thus, by using genetic algorithm method, it has shown that the ride comfort of bus is improved compared to when using the experimental parameters.

**Figure 9:**
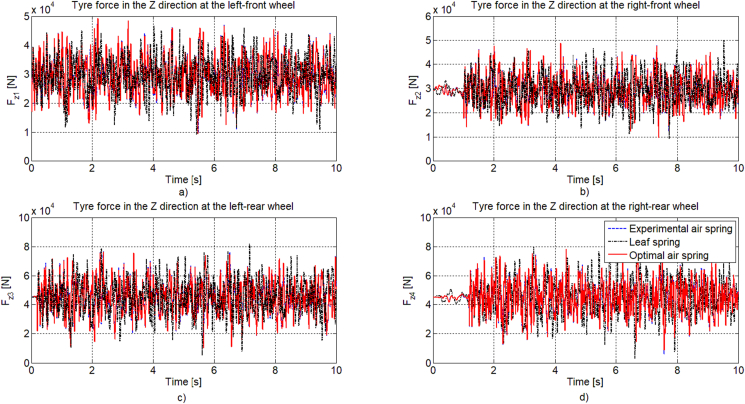
Time response of tyre force in the vertical direction: a) left-front (F_z1_), b) right-front (F_z2_), c) left-rear (F_z3_), d) right-rear (F_z4_) wheels.

Figure 9 shows the time response of the tyre forces in the vertical direction at four wheels, it can be easily seen that the difference in amplitude magnitude values of the two cases using the air suspension systems is not much different. This represents the optimal level of the road safety when designing and selecting the air spring element of the suspension system of the real bus.

**Figure 10:**
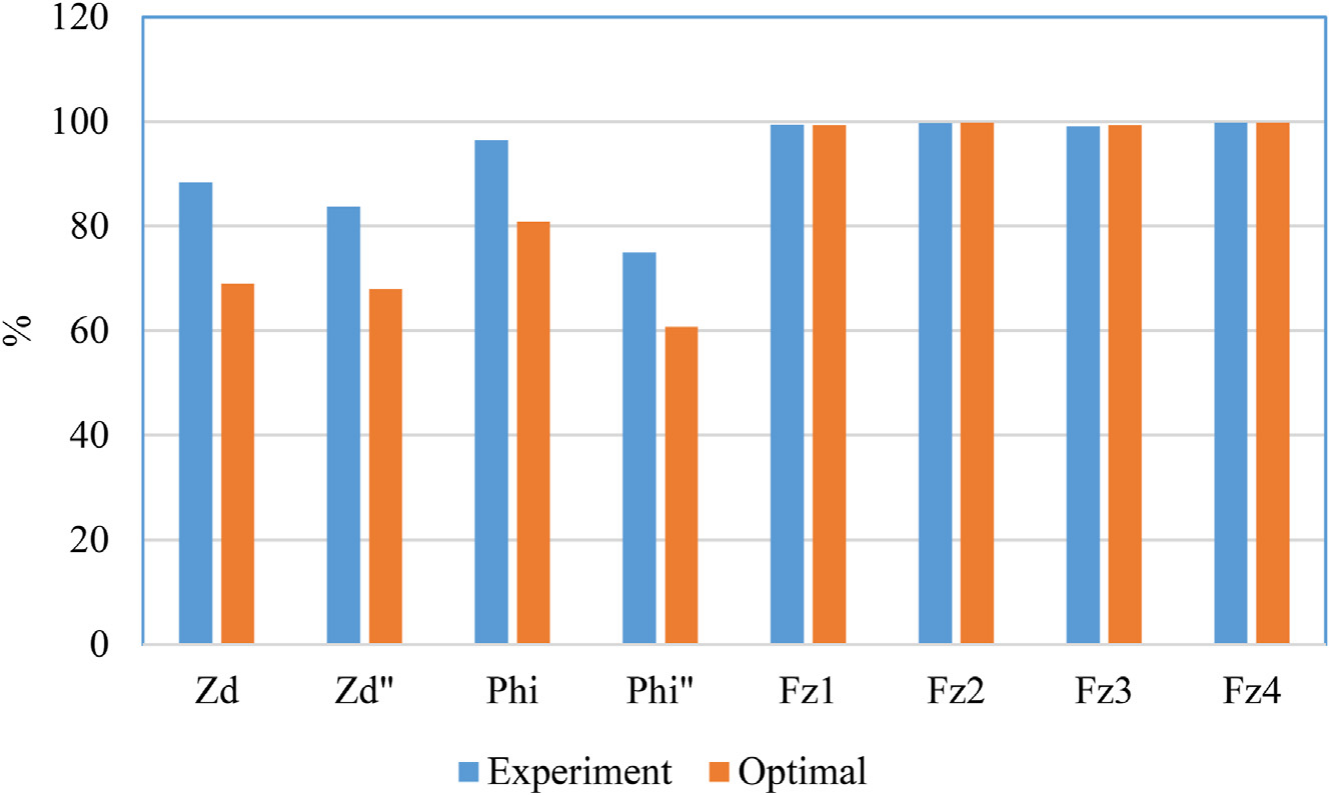
The comparison of the RMS value of the variables related to the variation criteria.

In order to evaluate the overall difference of results when using the two types of the air suspension systems with the leaf spring suspension system, the root mean square (RMS) value is compared as shown in Figure 10. Here the corresponding RMS value of variables related the leaf spring suspension system is assumed to be 100%. From this comparison diagram, it can be seen that the road safety of the bus using the air suspension system increases by about 2% through the RMS value of the tyre forces. However, the magnitude of the ride comfort is clearly improved about 5%–18% with the experimental air suspension system [9, 26, 32, 35, 36] and about 20%–40% with the optimal parameters from genetic algorithm method. This result is also compatible with the position of the experimental result according to the two optimal objectives shown in Figure 7. It can be seen that the air spring element used on real bus and tested has guaranteed relatively closing to *α* = 0.5. Therefore, the optimization problem with *α* = 0.5 has allowed to improve the ride comfort, meanwhile the road safety is still guaranteed.

## 6 Conclusions

The air suspension system is widely used in buses and heavy vehicles. This paper introduces the application of the genetic algorithm method to optimize the parameters of the air spring element in the air suspension system. Initially, the multi-objective optimization problem is introduced. The next step is to build a full vertical bus model using four air spring elements. The genetic algorithm method is considered to be used to optimize seven parameters of the air spring element. Optimal results are determined for each point on the Pareto curve. The simulation results in the time domain clearly show the advantages of the proposed optimal solution. Through choosing the value of the unique parameter *α*, the goal objectives are reasonably converted between the ride comfort and the road safety. This is clearly shown when compared with the experimentally determined parameters, by using the optimal parameters from the genetic algorithm method with *α* = 0.5, the ride comfort is improved by about 15%, meanwhile the road safety is still guaranteed.

Further research that can be developed is to find a solution to convert between optimal parameters when the bus is in different motion modes and there is a continuous transition between the road safety and the ride comfort. Moreover, the application of other optimization methods for the fully-integrated bus model is also a potential research direction.

## Declarations

### Author contribution statement

Truong Manh Hung: Conceived and designed the experiments; Performed the experiments; Analyzed and interpreted the data; Contributed reagents, materials, analysis tools or data; Wrote the paper.

### Funding statement

This research did not receive any specific grant from funding agencies in the public, commercial, or not-for-profit sectors.

### Data availability statement

The authors do not have permission to share data.

### Declaration of interests statement

The authors declare no conflict of interest.

### Additional information

No additional information is available for this paper.
